# Patients’ experiences of pharmacists in general practice: an exploratory qualitative study

**DOI:** 10.1186/s12875-021-01393-0

**Published:** 2021-03-05

**Authors:** Georgios Dimitrios Karampatakis, Nilesh Patel, Graham Stretch, Kath Ryan

**Affiliations:** 1 Peninsula Medical School, Faculty of Health, University of Plymouth, Drake Circus, Plymouth, PL4 8AA UK; 2 School of Pharmacy, University of Reading, Whiteknights Campus, PO Box 226, Reading, RG6 6AP UK; 3 Ealing GP Federation, 179C Bilton Road, Perivale, Greenford, Middlesex, UB6 7HQ UK

**Keywords:** Pharmacists, General practice, England, Patients, Experiences, Qualitative research

## Abstract

**Background:**

Since 2015, pharmacists have been integrating into English general practices and more recently into primary care networks. General practice-based pharmacists provide a range of patient-facing services, such as medication reviews, management of long-term conditions and minor ailments, prescribing duties and answering queries over the telephone. Literature reports patients’ satisfaction with general practice-based pharmacists’ services, however, previous research captured only limited experiences. The aim of the current study was to pursue an extensive exploration of patients’ experiences of pharmacists in general practice.

**Methods:**

General practice-based pharmacists, working in practices in West London, Surrey and Berkshire, handed invitation packs to patients seen during consultations. Patients that wanted to take part in the study were invited to undertake a qualitative, in-depth, face-to-face, semi-structured interview within the practice with which each patient was registered. Interviews lasted from 15 min to more than 1 h and were audio-recorded. Recruitment continued until data saturation. Audio-recordings were transcribed verbatim and transcripts analysed thematically.

**Results:**

Twenty participants were interviewed. Four themes were discerned: awareness (“I had been coming to this practice for 24 years and I didn’t know that there was a pharmacist”); accessibility (“People ring for a GP [general practitioner] appointment … it’s Monday and they [receptionist] tells you ‘We can slot you in on Friday’ … with a pharmacist on board, they can [instantly] look at you”); interactions (“I’ve always had a really good interaction with them [pharmacists] and they listen and they take on board what I’m trying to say”); and feedback (“It’s easier [to collect feedback instantly] because I could have forgotten half of what they [pharmacists] have told me in an hour or so’s time”).

**Conclusions:**

Findings indicate that pharmacists’ integration into general practices could improve accessibility to, and the quality of, care received. The findings will assist policy development to provide general practice-based pharmacists’ services as per patients’ needs.

**Supplementary Information:**

The online version contains supplementary material available at 10.1186/s12875-021-01393-0.

## Background

English general practices have been facing ongoing workload pressures stemming from an ageing population and reductions in the general practitioner (GP) workforce [1]. As a result, patients have been experiencing decreased access to primary care services, which has subsequently led to high levels of dissatisfaction [2–4]. To tackle these problems, and in parallel exploit the increasing numbers of qualified pharmacists [5, 6], there has been a wide drive to integrate pharmacists into general practices. Efforts to integrate pharmacists began with a two-phased scheme between 2015 and 2019, supported by the National Health Service (NHS), that introduced approximately 1000 general practice-based pharmacists in England [7]. In early 2019, the NHS Long Term Plan was announced that urged general practices to form Primary Care Networks (PCNs) [8]. PCNs are collaborative entities linking primary care services with hospital, social care and voluntary sector organisations and covering populations between 30,000–50,000 people [9]. PCNs are expected to hire about 26,000 additional staff by 2023/24, including large numbers of pharmacists, with employment costs fully reimbursed by NHS England [10]. Each of the approximately 1260 PCNs is expected to have at least one pharmacist by 2020 [11]. The projection is that by 2023/24, a typical PCN will have about five pharmacists, raising the total number of general practice-based pharmacists across England to about 7000 [12, 13]. A typical practice serving 10,000 patients is anticipated to have a pro-rata coverage by a pharmacist for 12 h per week [12].

Official statistics from NHS England show that in September 2020 there were 1582 full-time equivalent general practice-based pharmacists in England compared to 1249 in September 2019, which translates to an increase of 26.7% [14]. Despite the increase in the total number of general practice-based pharmacists, approximately 50% of the general practices in England did not have a pharmacist in early 2020 [15]. In addition, only half of the PCNs recruited a pharmacist in 2019 [16]. By the end of June 2020, 24% of the PCNs were still to claim funds from NHS England to hire a pharmacist [17]. The proposed reasons as to why a significant proportion of PCNs have not recruited a pharmacist included insufficient numbers of appropriately qualified pharmacists for the posts, low pay grades that discourage pharmacists from taking posts in PCNs, uncertainty of PCNs on how to effectively use the skills of a pharmacist and the recent pandemic [15, 17, 18]. As a result, there are claims that the expected targets with regards to general practice-based pharmacists’ numbers might be impossible to be achieved [15].

Common roles of English general practice-based pharmacists include face-to-face clinics with patients for structured medication reviews and long-term condition management; telephone consultations for minor ailments and triage; prescribing duties, for those qualified; and supporting staff in medication-related queries and meeting targets of incentive programmes [19–21].

Several countries have attempted to implement general practice-based pharmacists’ services, including Australia [22], Canada [23], USA [24], New Zealand [25] and the Netherlands [26]. In the UK, having pharmacists in general practice is not an entirely new concept [27, 28]. This is the first time, however, that the role is being implemented to a large extent and so needs to be formally tested [29]. Little is known about how the presence of pharmacists in general practice impacts the wider healthcare system, including patients and healthcare professionals, and there have been ongoing calls for a thorough exploration of the role [30–32].

Existing literature, both nationally [33–39] and internationally [40–45], has offered some insights into patients’ views of general practice-based pharmacists. Some of these studies elicited opinions before patients had any contact with a general practice-based pharmacist [44, 45]. Of the studies referring to views post contact with a pharmacist [33–43], most described the contentment of patients with pharmacists’ presence in general practice as well as intentions to recommend pharmacists’ services.

Previous research efforts in England, however, were conducted some years ago and were limited to specific geographical regions, hence unlikely to have accounted for the whole range of employment models and roles of general practice-based pharmacists. Additionally, the waves of pharmacists still being integrated into English general practices might translate to varying patients’ experiences as a result of increasing exposure to pharmacists’ services as well as to diverse skillsets of pharmacists. The aim of the current study was to pursue an exploratory approach and explore patients’ experiences of general practice-based pharmacists in three different locations in England and therefore contribute to a more universal mapping of experiences. In particular, the current study set out to answer the following research question: What are the patients’ experiences and views of pharmacists working in general practice?

## Methods

### Study design

A realistic qualitative interview design was chosen to allow for an in-depth exploration of experiences, using interpretive thematic analysis.

### Setting

Participants were recruited from large general practices located in West London, Surrey and Berkshire, targeted as recruitment points due to working connections with the research team’s organisation. The West London practice, with a list of approximately 16,000 registered patients, has participated in the ‘pharmacists in general practice’ scheme since inception. This practice was composed of two sites and, at the time of the study, had 12 GPs, four nurses and three pharmacists. Pharmacists (in total) served approximately 170 patients per week, both through face-to-face and telephone sessions. Practices in Surrey and Berkshire were not part of the initial scheme but at the time of data collection they also employed pharmacists. The practice in Surrey was composed of a single site, had 16,000 patients registered and 13 GPs, two trainee GPs, five nurses and one pharmacist. The pharmacist dealt with approximately 100 patients on a weekly basis (both face-to-face and over the telephone). The practice in Berkshire consisted of two sites, had 14,000 registered patients and employed nine GPs, two nurses and two pharmacists. Pharmacists there served approximately 60 patients per week in total, both via face-to-face and telephone appointments.

### Recruitment

A purposive sampling approach was followed to recruit people who have visited a pharmacist in general practice. General practice-based pharmacists, working in the above-mentioned practices, handed invitation packs to eligible patients they met during consultations. Patients were eligible if they were aged 16 years or over, English speakers and able to consent for themselves as determined by the recruiting pharmacist. Invitation packs contained the study’s invitation letter; information sheet, providing details of the study and research team; consent form; reply form; and a business reply envelope. The study’s documents asked potential participants to directly contact a member of the research team (GDK), either via email or by filling in the reply form and posting it within the pre-paid envelope. GDK is a doctoral research student with experience in qualitative research. Once interest for participation was expressed, GDK contacted potential participants and a mutually convenient time for the interview was arranged. No other reminders were sent. Recruitment continued until data saturation. The research team interpreted data saturation as the point in data collection at which no new information was discernable, also known as ‘informational redundancy’ [46–48]. When this point was reached, four more interviews were conducted before recruitment ceased.

### Data collection

Recruitment and data collection took place between December 2018 and February 2020. Involvement in the study was voluntary and without monetary incentives. Audio-recorded interviews were conducted by GDK in private meeting or consultation spaces within the general practice with which each participant was registered. Just before each interview, mutual introductions took place to establish rapport and any questions that participants had were answered. In addition, the confidentiality of the interviews was highlighted by emphasising that discussions could not be overheard by general practice-based pharmacists, that findings would only be discussed between the research team without any disclosure to pharmacists and that any data to be used in research outputs would be anonymised. All interviews were face-to-face and semi-structured. An interview schedule, consisting of some open-ended questions and prompts, was used. The interview schedule was developed exclusively for this study and can be found as an additional file (see Additional file 1). Interviews terminated only when participants did not have anything else to add. Interviews lasted from 15 min to more than 1 h. Demographic information was collected at the time of interview.

### Data analysis

Audio-recordings were transcribed verbatim half by GDK and half by a professional transcribing agency, a sub-set of the latter was checked for accuracy by GDK. Transcripts were analysed thematically by following the steps of Braun and Clark (data familiarisation, data coding, identifying themes, re-examining themes, defining and naming themes and synthesising the report) [49]. Data was inductively [50] coded by GDK with the aid of NVivo 11 software, and a single code was ascribed to every different idea. Coding was verified by the whole research team via debriefing meetings in which thorough discussions took place. Data under the same code was collated together and sorted into categories, which were then re-examined and collapsed into possible themes with associated subthemes. The research team collectively assessed, refined and named the themes, again during debriefing meetings. Participants’ feedback on transcripts or findings was not sought.

## Results

Twenty participants were interviewed in total. There was an equal proportion of male to female participants. All had some contact with a general practice-based pharmacist. Participants were from different age-groups but all were aged 40 years or older. Most were from a white British and other white backgrounds. Table 1 provides an overview of participants’ demographics.

**Table 1:** Demographics of participants

	Age-group (years)	Number of visits to the pharmacist in general practice*	Ethnicity	Location from where participants were recruited
**Patients (*n*** **= 20)**	40–49 (*n* = 2) 50–59 (*n* = 5) 60–69 (*n* = 5) 70–80 (*n* = 6) 80+ (*n* = 2)	1 to 12 times	White British (*n* = 13) White Irish (*n* = 3) Other White (n = 2) Arab (*n* = 1) Other Asian (*n* = 1)	West London (*n* = 7) Surrey (*n* = 9) Berkshire (*n* = 4)

*This does not include contact over the telephone

### Themes

**Fig. 1:**
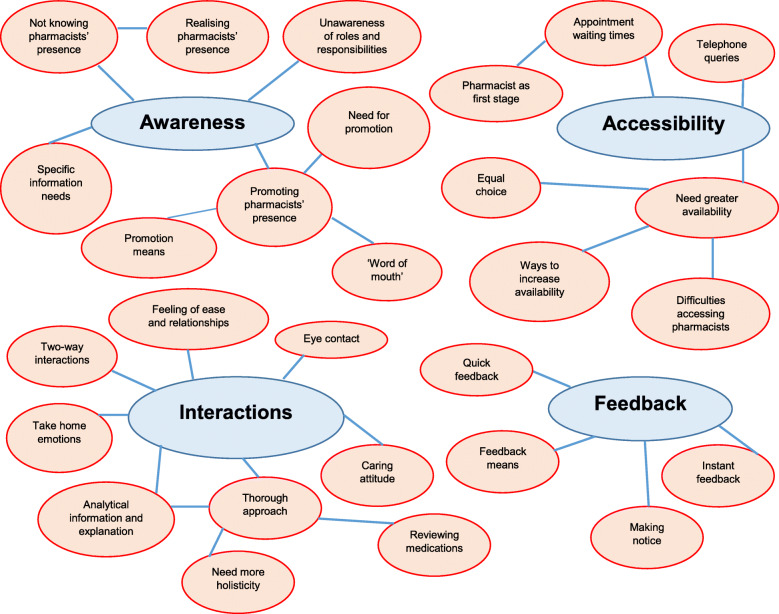
Themes and subthemes of patients’ experiences of pharmacists in general practice

Four predominant themes were discerned in the data: awareness; accessibility; interactions; and feedback. Figure 1 provides an overview of the themes and associated sub-themes.

### Theme 1: awareness

Most participants claimed that patients were largely ignorant of the presence of pharmacists in general practice due to a perceived absence of relevant information material. They all realised by chance the existence of general practice-based pharmacists, just before or only at the time of consultation.

*I had been coming to this practice for 24 years and I didn’t know that there was a pharmacist here. It’s possibly not my fault, they don’t advertise, promote, they don’t explain enough … I got a text [message] saying “make an appointment with the pharmacist” … [I was thinking] “What are they talking about”? “Where”?* (Participant 5)

There was also uncertainty, and often confusion, about the roles of general practice-based pharmacists and whether or not contact would be ongoing.

*I still don’t know why a patient would want to see a pharmacist in the general practice. I can’t get my head around that. ‘Cause if I want to ask the pharmacist something I go into the actual pharmacy. I’m not aware of the full extent of what they do.* (Participant 10)

Nearly all participants emphasised the need to promote the presence of pharmacists in general practice, to raise patients’ awareness and therefore encourage the uptake of pharmacists’ services. Numerous methods of promotion were proposed, such as television advertisements; messages on practice websites, social media accounts, waiting room screens and noticeboards, including introducing the pharmacist amongst the healthcare team photographs; posters and leaflets; and establishing visible consultation spaces for pharmacists.

Word of mouth was largely seen as an effective way to promote services, including through outreach activities and referrals.

*I have three housemates registered in this practice and I can spread it by mouth, “You know that there’s a pharmacist that attended to me today, they helped me a lot by giving me information”.* (Participant 13)

*If a GP feels “well, this particular problem would be better decided by a pharmacist” [then they should] refer. But that doesn’t seem to happen very often.* (Participant 20)

Apart from the existence of general practice-based pharmacists, additional information needs of patients included the specific services offered by pharmacists; reasons to contact pharmacists; what is outside the pharmacist’s remit; and the potential benefits of seeing the pharmacist.

### Theme 2: accessibility

General practice-based pharmacists were perceived, by several participants, to be readily available to take patients’ queries over the telephone, in contrast to GPs who were much more difficult to contact.

*The receptionist says “Oh yes, the [pharmacist is] in today. I’ll just ask them to chat to you”, and it’s done within a day. Or the reception will say, “They’re not in today but they’ll be in tomorrow and I’ll get them to call you”.* (Participant 15)

Similarly, some participants claimed that there was far less waiting time with pharmacists’ appointments, both with scheduling an appointment and in the waiting room. There were some suggestions that seeing the pharmacist should become the first stage when an appointment at the practice is required.

*People ring for a GP appointment and they can be at death’s door … [and] it’s Monday and they [receptionist] tells you “We can slot you in on Friday”. With a pharmacist on board, they can [see] you and if it’s something more serious they would speak to the doctor. It’s a faster system … you could have an 11 o’clock appointment for a GP and you won’t be seen until 12-12.30. With the pharmacist, it may run over five minutes, it may be ten minutes but no more than that.* (Participant 1)

A few participants, however, reported occasional difficulties in getting appointments with pharmacists covering multiple practices, due to reduced availability and/or uncertainty about the exact days pharmacists were present in a specific practice. Likewise, one participant was frustrated not to directly be put through to the pharmacist, as phone calls were often returned at inconvenient times. Another one complained about pharmacists sometimes cancelling their appointments last moment. Some participants called for larger numbers of pharmacists, weekend sessions, appointments on the same day as GP appointments and availability of drop-in clinics.

Some participants mentioned that contact with the pharmacist should always be offered as a choice to the patient, in triage and online booking systems.

*[Seeing] the pharmacist should be an equal opportunity [to the GP], a choice for patients. Even if you went through a telephone screening … [also] to have an online booking system which would incorporate the pharmacist.* (Participant 3)

### Theme 3: interactions

The vast majority of participants emphasised the high quality of the interactions they had with general practice-based pharmacists. They reported that their consultation with a pharmacist was a two-way interactive process. Pharmacists were believed to treat patients as equal fellow-speakers, rather than passive recipients of instructions, and to welcome patients’ thoughts and questions.

*I have colitis [and] I have a suspicion that it is triggered by sugar. I tried to have a discussion with the doctor but they didn’t want to discuss it, they just said, “There is no research on that at all”. At my last meeting with the pharmacist here, I mentioned it to them. They had a really useful discussion with me about it. And I came away feeling that I had been listened to. I felt that I had an informed and adult discussion. With the doctor, often they treat you like children “the doctor knows best, this is what you’re gonna [do]”.* (Participant 2)

The perceived absence of hierarchies and judgemental approaches by nearly all participants made them feel at ease with pharmacists and established mutual familiarity and relationships.

*Well sometimes when you come to the doctor, I am always conscious of the time and I don’t waffle. I don’t just waste the doctor’s time … So, there’s a certain level of anxiety, stress ... I found that with the pharmacist there was a less judgemental attitude, they were very approachable, immediate, very easy to talk to.* (Participant 6)

*The pharmacist, I’ve seen them once and I feel like I’ve known them for a long time. That’s unusual ... I know the pharmacist’s name. That’s the difference. I’ve seen loads and loads of doctors here, more than once, and I don’t know their names.* (Participant 3)

Pharmacists, several participants claimed, visually connected with patients during consultations, which they took as an expression of being paid attention to.

*They [pharmacist] looked me straight in the eye and I think sometimes if you’re not looking at someone, you’re probably wandering with your mind, whereas, they were concentrating on me.* (Participant 12)

Many participants emphasised the caring attitude of pharmacists, which they attributed to fewer time-constraints compared to GPs.

*They [pharmacists] are very dedicated in what they do, they’re empathetic … the doctors, once the ten minutes are up, they stand up [and] you haven’t even finished and you have to go. I hate that. I think that’s dreadful.* (Participant 8)

Several participants claimed that, as a result of longer appointment times, pharmacists were thorough in their approach, including concomitantly managing multiple co-ailments and developing structured care plans.

*I had a new set of blood tests done which showed that my cholesterol levels had increased … They [pharmacist] went through my lifestyle, diet, exercise, where I live … They were thorough … [and] set a good plan to go forward, [to] have a review after three months and see how we go … I [also] had some twitching in my calf muscle, they weren’t quite sure what it was, so they saw one of the GPs in the practice and chased it through with them.* (Participant 7)

One participant, however, was afraid that pharmacists occasionally exceeded competency and requested more referrals to specialist care.

Pharmacists were reported, by several participants, to always review every single medication patients had, regardless of whether they related to the presenting complaint.

*The [pharmacist] went through my list of other medications [as well] and dismissed the ones that I didn’t really need to keep on my repeat because I wasn’t having them … They said “Let’s take them off because if you do need them in the future, they can be put back on again”. No GP has ever said that to me before. So, you can see how older people just have this long list of medications that they may, if they don’t realise, still be taking.* (Participant 17)

Nearly all participants emphasised the information/explanation that pharmacists provided, including the analytical way this was conveyed, which was seen to allow patients to fully understand their medication or condition and convince them to accept the pharmacist’s advice.

*The [pharmacist] has given me some reading material to take away with regards to possible injection [for my diabetes]. They showed me with a dummy pen how it would be administered. So, yeah, it was very informative. They took the time to actually explain [everything] … the book of information, they took the time to actually go through the pages, give a brief outline, how it may or may not influence me.* (Participant 14)

Conversely, a few participants stated that pharmacists should consider potential side effects of medications upfront (rather than trailing different medications) and explain everything about medications without having to be prompted. One participant mentioned that pharmacists should also consider alternative therapies, such as natural substances and homeopathic remedies. Another participant was disappointed about pharmacists not recording condition−/medication-related history, hence having to re-provide these details in subsequent consultations.

All participants left consultations with pharmacists feeling confident, reassured and with peace of mind that their problems had been resolved.

*My fear was that the medical people were going to keep pushing statins at me, regardless of my side effects. But they [pharmacist] said, “Look, we won’t try any more”. So, I was reassured by the fact that I’m not going to be pushed statins forever and I feel completely reassured that my interests are being properly looked after in terms of prescribing medication.* (Participant 11)

*A lot of doctors made me feel quite a hypochondriac … with the pharmacist, you feel a sense of security after leaving them.* (Participant 1)

### Theme 4: feedback

Some participants doubted if patient feedback on general practice-based services was taken seriously into consideration.

*Usually the feedback, the result of that doesn’t go back to the people … Is that [feedback] making any difference, is that making any improvement? Did anybody read it? Has it been put in practice?* (Participant 4)

There was no consensus amongst participants on the preferred way to collect patient feedback on general practice-based pharmacists. Various means were proposed, such as face-to-face interviews; questionnaire forms, either as hard copies or online, including using tick-boxes or rating scales or human faces mirroring satisfaction level; and politely reporting concerns directly to pharmacists. Most participants stated that the overall process of feedback provision should be quick, to encourage participation. As such, participants claimed, any feedback collection tool should be short in length.

The majority of participants also stated that feedback should be collected straight after consultations.

*It’s easier [to collect feedback instantly] because I could have forgotten half of what [the pharmacist] told me in an hour or so’s time. I’d go “What did they say about my tablet”? … So, [it would be good] to get at me [for feedback] quickly afterwards, while I remember things.* (Participant 19)

*The public wants [to give feedback] right at the time they are having the consultation … because if they give [a form] to you, then you go back to your office and you set it down and two or three days later you have more things piled up and you never send the feedback.* (Participant 5)

## Discussion

Findings indicate that patients are unaware of pharmacists’ presence in general practice and/or unclear when to contact pharmacists. When they do interact with general practice-based pharmacists, however, patients highly appreciate the quality of care they receive. Some ways to enhance the availability of pharmacists and collection of patient feedback were suggested.

The findings of the current study could best be interpreted in light of ‘scientific realism’, which views ‘realities’ in the contemporary world as meanings constructed by human minds [51, 52]. The key feature of ‘scientific realism’ is the element of explanation, which is illustrated in the slogan question of ‘what it is within a programme that works or does not work well, for whom and under what circumstances’ [52, 53]. The correlation between ‘scientific realism’ and our findings lies in the fact that the current study identified strengths and limitations with pharmacists’ presence in general practice, as viewed by patients, through an exploratory approach that sought to understand in-depth the reasons of why certain aspects with pharmacists in general practice work or do not work for patients.

More simply, elements with pharmacists’ presence in general practice that ‘work’ for patients include pharmacists’ availability, providing that pharmacists do not cover many practices and patients consciously seek pharmacists’ care; and the high standard of interactions, which lead to positive emotions and a strong relationship between patients and pharmacists, and occur when there are no time-pressures during appointments. In contrast, the aspect with pharmacists in general practice that ‘does not work’ for patients is the existence of multiple information needs, due to the absence of relevant information, which limit the uptake of pharmacists’ services.

Below, findings are related to pre-existing literature whilst also taking into account current ‘social’ circumstances which could affect patients’ satisfaction with aspects of pharmacists’ presence in general practice.

### Comparison with existing literature and realistic discussion

The current study highlights the limited awareness of pharmacists in general practice amongst patients. Previous UK research has also reported unawareness due to absence of relevant communication from practices, including patients not realising that they had a consultation with a pharmacist, and confusion between community and general practice-based pharmacists’ roles [34, 38]. Post publication of previous studies, our findings imply that there is still no clear direction (either at a central or local level) to inform the wider public about general practice-based pharmacists’ existence, what services they provide and how to access them.

Participants who consciously sought access to general practice-based pharmacists’ services found pharmacists more accessible than GPs, something that has also been widely reported in literature [34, 36–38]. Our findings indicate that pharmacists’ integration into general practices could fulfil the aim of offering patients smoother access to healthcare services and checks, however, the achievement of this goal is largely hindered by patients’ unawareness of the presence of in-house pharmacists in practices. Moreover, a few of our participants reported difficulties in accessing pharmacists covering multiple practices, a phenomenon also noticed by patients in Australia [42]. Open-ended questions remain about accessibility to pharmacists in the future. Firstly, because pharmacists in PCNs are expected to work across multiple practices [12, 21] and secondly, because as awareness improves and demand increases, consultation times are likely to decrease. In addition, increasing numbers of remote consultations over the telephone (following the coronavirus pandemic) [54] might restrict pharmacists’ ability to respond to patients’ queries.

Our findings around the long duration and thoroughness of pharmacists’ consultations repeat those of previous studies [33–42]. The novelty of our study, however, is that it also offers insight into the dynamics of interactions between patients and general practice-based pharmacists. These dynamics can be discussed using King and Hoppe’s ‘6-function model’ [55], which is a consensus-derived framework using six key functions to understand ‘good approach’ in patient-practitioner encounters (see Table 2 for an overview of the model).

**Table 2:** Overview of the ‘6-function model’, analysing ‘good approach’ in patient-practitioner interactions

Function of interaction	Brief description
Fostering the relationship	Refers to establishing rapport and connection between practitioner and patient.
Gathering information	Refers to collecting as much information as possible from the patient to understand their needs from the encounter.
Providing information	Refers to offering information to the patient to facilitate understanding.
Decision making	Refers to enabling patients’ deliberation and decision making, including developing action plans.
Enabling disease- and treatment-related behaviour	Refers to fostering self-management of the patient.
Responding to emotions	Refers to showing empathy and assisting patients in developing positive emotions.

In our case, the function of ‘fostering relationships’ was achieved by suppressing hierarchies/judgements and maintaining eye contact that established a welcoming environment for patients and generated mutual bonds. The function of ‘gathering information’ was illustrated by pharmacists’ keenness to “listen to the patient” and constant effort to collect details on condition or lifestyle or medications, thereby avoiding unwarranted conclusions and leaving patients feeling that they had been heard. ‘Providing information’ was mirrored in the detailed explanations, including using graphic and/or descriptive means. ‘Decision making’ was indicated by the absence of pressure on the patient to follow certain treatments and the development of structured care plans. ‘Enabling disease- and treatment-related behaviour’ was obvious in the better understanding of medications patients developed post contact with the pharmacist. The ‘responding to emotions’ function was obvious in the caring attitude and reassurance offered by pharmacists, and the opportunity for patients to speak freely and express their concerns. Therefore, all key functions of patient-centric communication were witnessed in our findings. The absence of time constraints contributed to the ability of pharmacists to interact at this high level during consultations. It is unclear through our findings, however, whether any skillset of pharmacists (different from those of GPs) also had some role to play.

Last but not least, our study offered insight into patients’ preferences with regards to feedback collection. To help action these insights, the patients could themselves be involved in determining what type of feedback is required, how it is collected and information disseminated. There is ample literature describing involvement of patients at various stages in research, such as in designing research priorities, questions, methods, protocols and study documentation as well as in data collection, analysis, interpretation and dissemination [56–60]. Patient and public involvement (PPI) often links with positive outcomes, such as practical improvements in healthcare services (ranging from informational material for patients to changes in the delivery of services and the behaviours of healthcare staff), increased participation rates in studies and additional layers of understanding of research data [56, 58, 61–64]. Despite the described benefits, PPI attempts in research are not extremely common in the general practice setting due to limited resources and fears of complicating projects [65]. However, patient participation groups (PPGs) in general practices are an easy way of accessing PPI and could be actively involved in designing and implementing a patient-friendly feedback mechanism on general practice-based pharmacists’ services. Our findings could act as a starting point to involve PPGs in feedback collection.

### Strengths and limitations

The qualitative design used in the current study allowed for an in-depth understanding of patients’ experiences. The study was carried out at general practice settings diverse in terms of location and integration of pharmacists, hence the experiences captured are reflective of some different models of employing pharmacists in general practice and varied exposure of patients to pharmacists. Despite the limitations of ethnicity and age in the sample (see below), sufficient data saturation was achieved to offer findings a conceptual depth. We are confident, therefore, that findings synthesise a wide (if not the whole) range of potential experiences of patients at the specific recruitment points. Findings primarily apply to the UK reality, however, individual elements will still be useful for international attempts to integrate pharmacists into general practices.

We did not set out to include a representative sample of the population in terms of age, ethnicity and region of domicile. Participants were recruited only from three practices in the south of England. As a result, findings might not be fully generalisable but can provide insights that could be extrapolated to other similar settings. Moreover, participants who volunteered for interviews mainly included white and older people, hence findings do not offer a good representation of younger age-groups and black and ethnic minorities who may have different experiences. It could be that older patients are most likely to face polypharmacy and other medication-related problems and so use pharmacists’ services, hence why younger patients were missing from our sample. The fact that interviews were carried out inside general practices might have introduced some biases in the responses of participants, due to a potential fear that pharmacists would learn about participants’ views. We believe, however, that the mutual trust established between the interviewer and participants and the reassurance about the confidentiality of the study encouraged the expression of honest views, hence making it very unlikely that findings would have significantly differed if interviews were carried out outside general practices. Although the research team followed a reflexive approach by ignoring personal experiences and collectively analysing data, because they are all pharmacists, some unavoidable instances of personal assumptions during data categorisation might still exist.

### Implications

The specific implications of the current study are that there is a need to:

- Appropriately educate patients and the public about general practice-based pharmacists, including roles and responsibilities.
- Ensure pharmacists are present in the practice for an adequate amount of time each week, ideally on a daily basis, and explicitly communicate rotas to patients by also establishing an effective triage system to prevent exhaustion of pharmacists’ resources.
- Secure the prerequisites for efficient interactions with patients, for example, adequate appointment lengths.
- Design a formal, quick and attractive feedback mechanism for patients.

Future studies should employ maximum variation sampling to include experiences of patients from different ethnicities, ages, educational levels and regions of domicile in the UK. Future studies should also include developing additional measures to more thoroughly explore the added value pharmacists bring in general practice settings and co-designing pharmacists’ services with the public, including developing interventions to satisfy information needs with regards to pharmacists in general practice.

## Conclusions

The current study indicates that pharmacists’ integration into general practice has the potential to enhance the timely access to, and quality of, services in primary care. Practitioners, including pharmacists themselves, can use our findings to enhance their own practice by improving patient-centred interactions during consultations. More importantly, findings will inform delivery of the NHS Long Term Plan on how to make best use, from a patient perspective, of general practice-based pharmacists and will also assist practices when attempting to promote the benefits of having a pharmacist. Results will also guide international policy about integrating pharmacists into general practices, including how to design and evaluate patient-centric services.

## Supplementary Information

**Additional file 1:.** Interview schedule. This additional file consists of a table that presents the interview schedule developed and used in this study.

## Data Availability

The datasets generated and analysed during the current study are not publicly available because that would compromise participants’ anonymity and the researchers are still publishing findings. Datasets, however, are available from the corresponding author on reasonable request.

## Abbreviations

GP: General Practitioner
NHS: National Health Service
PCN: Primary Care Network
PPG: Patient Participation Group
PPI: Patient and Public Involvement
