# Supplementary material for: Patients’ experiences of pharmacists in general practice: an exploratory qualitative study
Source: BMC Fam Pract. 2021 Mar 5;22:48. doi: 10.1186/s12875-021-01393-0 (PMC7935482; doi:10.1186/s12875-021-01393-0)
Supplement: Supplementary file 1 — Additional file 1:. Interview schedule. This additional file consists of a table that presents the interview schedule developed and used in this study. [file 12875_2021_1393_MOESM1_ESM.docx]

**Additional file 1. Interview schedule**

| **Intro (setting the stage)** | **Questions** | **Closing** |
| --- | --- | --- |
| - Introduce myself - Explain the aim of the study - Explain the process (highlight confidentiality) - Answer questions the participants might have - Ask participants to sign the Consent Form - Collect demographics - Explain that the word ‘service’ means consultation, or other means of contact, with a pharmacist in the surgery - Switch on the recorder (after gaining permission) – start the interview | - Tell me about your experiences of consulting the pharmacist in the general practice   Prompts:   1. What did you like about the service? 2. What you didn’t like about the service? 3. What can be improved with the service? How? 4. What additional expectations/needs do you have from the service? 5. What other preferences do you have in the way the service is offered (e.g. access to the service)?  - How do you think pharmacists in general practice could be better accepted by patients?   Prompts:   1. What do you know about patients’ awareness of the service? 2. How should the service be best promoted to patients?  - Tell me about how would you prefer to give feedback on the service?   Probes:   - Please give me an example of that. - Please tell me a bit more about that. - Please be a bit more specific about that. - What do you mean by that? | - Ask participants if they have anything else to add (then stop recording) - Inform them how they can reach the research team if they have more questions - Thank them – let them know how helpful they have been |
